# Litholapaxy

**Published:** 1881-11

**Authors:** Bernard Bartow


### Litholapaxy.

Reported by Bernard Bartow, M. D.

In June of the present year, Mr. ——, æt. 66, came to me to be treated for frequent micturition, difficult and painful in character. About twenty-five years ago he underwent a course of treatment for urethral stricture; since that time the stricture had received no attention. He had had two attacks of renal colic—one in January and one in April of the present year. After the first attack a small calculus, the size and shape of an oat, escaped *per urethram.* Examination of the urethra showed constrictions to be present in the spongy and membranous portions, the former admitting No. 17 sound, the latter No. 11 (French). In four weeks, by gradual dilation, the calibre of the canal was so restored as to permit the passage of No. 30 sound. In the course of the treatment there escaped through the urethra a phosphatic calculus the size of a small bean. After removal of the obstruction to urination there did not follow the improvement in the patient's condition that was expected. The more perfect contraction of his bladder that he could then exert developed symptoms of vesical calculus, of which there had been no clear indications previous to that time. Examination of his bladder for calculus was twice made, and in each instance without success in finding one.

A third exploration of his bladder was made October 8, when a calculus was detected; measurement by the searcher did not show it to be of large size. Owing to the age and physical condition of the patient, the operation of litholapaxy was chosen.

In view of their dilatable character, the existence of strictures was not considered a contra-indication. As a preparatory measure, a No. 30 sound was introduced into his urethra, which permitted the sound to pass into the bladder without resistance.

Oct. 13, the patient having been etherized, a lithotrite with flat blades was passed into his bladder and the calculus freely crushed. After the withdrawal of the instrument, the meatus was enlarged and a No. 30 straight evacuating tube introduced into the urethra. It was found impossible, however, to advance it beyond the stricture, in the membranous portion; a curved tube of the same calibre was similarly obstructed at that point Smaller tubes not being at hand, evacuation of the fragments was temporarily deferred. The patient was one and one-half hours under the influence of ether. Forty minutes were occupied in crushing the calculus, the diameter of which, as shown by the scale of the lithrotrite, was 28 c. m. Assistance was rendered by Drs. Hopkins, Folwell, Gay and Mynter.

Two grains of opium in suppository and five grains quinine *per orem* were administered as soon as the patient had sufficiently recovered from the anæsthetic.

No constitutional or local disturbance was induced by the operation, nor did any develop in the following thirty hours. Urination was accompanied by none of the suffering that attended it prior to the operation. Small quantities of *debris* escaped with each emission of urine. After thirty hours a No. 28 evacuating tube was obtained and was introduced into the patient's bladder with ease. The aspirator was attached, and what afterward proved to be one-third of the fragments of the calculus, removed. This part of the operation was done without an anæsthetic, and caused the patient great pain, followed by a severe rigor about an hour afterward. As the amount of the *debris* removed did not correspond with the size of the stone, it was evident that there were fragments remaining too large to escape through the tube. As their presence induced no irritation of his bladder, he was allowed three days' rest.

On the fourth day the operation was repeated. Several large fragments were crushed and evacuated, in amount, as afterward shown, about one-third of the calculus. The time occupied by the operation was one hour. No shock to the patient ensued. A more normal condition of the urinary functions was noticeable after the removal of the fragments.

Nine days from the time of first operation a thorough exploration of the patient's bladder was made with a lithotrite. Several small pieces of the stone were crushed and evacuated, and the search continued until it was evident that nothing remained to form the nucleus of another calculus. The amount removed on this occasion with that which escaped at previous urinations completed the remaining one-third of the quantity of *debris* that was collected. On this occasion, also, ether was employed, and its effect maintained one hour. The composition of the calculus is phosphatic, the dried fragments weighing 4.29 grams.

Two days after the last operation the patient was able to resume his business. His relief has been complete, and with the exception of slight debility, no unpleasant effects remain from the operations. On several occasions pieces of stone became lodged in the prostatic and membranous portions of the urethra, causing intense pain when attempts were made to dislodge them with forceps or bougie. It was feared that abscess might result from their non-removal. Relief from them was obtained in the following manner: A soft rubber catheter (the end of which had been cut-off above the eye so as to give it an open extremity) was introduced to a point a little in front of the fragments. The urethra was then compressed upon the catheter with the hand, and a current of water forced through the catheter into the bladder; this dislodged the pieces and washed them back into the bladder, from which they were afterward removed through the tube. When the urine showed a tendency to rapidly decompose, the bladder was injected every 8 hours with 1 per cent. sol. carbolic acid; it ceased to be ammoniacal in twelve hours after the complete evacuation of the calculus. The only instance of shock to the patient in the whole course of his treatment was on the occasion when evacuation was undertaken without anæsthesia. With that exception the progress of the case was unattended by a symptom of systemic irritation.

